# Associated Factors to Seroprevalence of* Ehrlichia* spp. in Dogs of Quintana Roo, Mexico

**DOI:** 10.1155/2016/4109467

**Published:** 2016-12-21

**Authors:** Pedro Pablo Martínez-Vega, Manuel Emilio Bolio-Gonzalez, Roger Iván Rodríguez-Vivas, Eduardo Gutierrez-Blanco, Carlos Pérez-Osorio, Sandra Luz Villegas-Perez, Carlos Humberto Sauri-Arceo

**Affiliations:** ^1^Laboratorio de Parasitología, Centro de Investigaciones Regionales “Dr. Hideyo Noguchi” Unidad Inalámbrica, Universidad Autónoma de Yucatán, Calle 96 S/N Cruzamientos Av. Jacinto Canek y Calle 47, 97225 Merida, YUC, Mexico; ^2^Departamento de Salud Animal y Medicina Preventiva, Facultad de Medicina Veterinaria y Zootecnia, Universidad Autónoma de Yucatán, Km. 15.5 Carr. Mérida-Xmatkuil, AP 4-116 Itzimná, Mérida, YUC, Mexico; ^3^Unidad de Investigación Clínica y Epidemiología, Facultad de Medicina, Universidad Autónoma de Yucatán, Av. Itzáes No. 498, 97000 Mérida, YUC, Mexico

## Abstract

The objective of this study was to determine the seroprevalence to* Ehrlichia* spp. in dogs from Xcalak, Quintana Roo, Mexico, and the associated factors. Serum samples were obtained from 118 dogs and used in an indirect immunofluorescent assay test for the detection of antibodies against* Ehrlichia* spp. A questionnaire was used to obtain information about possible variables associated with seroprevalence. These variables were analyzed through Chi^2^ test and logistic regression. Dog seroprevalence of antibodies against* Ehrlichia* spp. was 64% (75/118). Fifty-two percent (61/118) of dogs had tick infestation which was identified as* Rhipicephalus sanguineus sensu lato*. Anemia was observed in 36% of dogs. Leucopenia (2.5%), thrombocytopenia (70%), and hemorrhage (14%) were also observed. Thirty-one percent (23/75) of dogs with anemia, 4% (3/75) of dogs with leucopenia, 80% (60/75) of dogs with thrombocytopenia, 17% (13/75) of dogs with hemorrhages, and 59% (44/75) of dogs with ticks were positive for* Ehrlichia* spp. antibodies. The factors associated with seroprevalence were age (1–3 and >3 years old, OR = 7.77 and OR = 15.39, resp.), tick infestation (OR = 3.13), and thrombocytopenia (OR = 3.36). In conclusion, seroprevalence of* Ehrlichia* spp. was high in the community of Xcalak and its associated factors were age, tick infestation, and thrombocytopenia.

## 1. Introduction

*Ehrlichia canis* (*E. canis*) is the most important species of* Ehrlichia* found in dogs; however,* Ehrlichia chaffeensis* (*E. chaffeensis*) and* Ehrlichia ewingii* (*E. ewingii*) may cause a clinical illness on this animal species [1–4]. The disease in dogs is classified as acute, subclinical, or chronic, based on the chronological appearance of clinical signs and pathological findings [5–7]. These three pathogen species belong to Anaplasmataceae family and order Rickettsiales. They have a 97% similarity in their 16S rRNA sequence and they also share similar immunogenic epitopes [8–13]. Therefore, it is not uncommon to observe cross-reactions in serological tests among members of this genogroup [2, 9, 14]. The above observation was demonstrated by several studies in which* E. canis* antigen was used in serological assays to determine exposure to* E. chaffeensis* in humans and* E. ewingii* in dogs [15–18]. The three* Ehrlichia* species have the potential of zoonotic transmission through vectors (monocytic canine ehrlichiosis, human monocytic ehrlichiosis, and canine granulocytic ehrlichiosis); although the role of the dog is not clear yet in the epidemiology of the disease in humans [2, 19–21]. The distribution of ehrlichiosis correlates with the presence of the vector [20, 22]. The tick* Rhipicephalus sanguineus sensu lato* is the main vector of* E. canis*, but* E. chaffeensis* and* E. ewingii* DNA have been detected also in this tick species [23, 24]. Ehrlichiosis is considered endemic in tropical and subtropical regions since these areas present adequate climatic conditions for the tick vector growth and development [22, 25]. The disease in dogs has been reported in Mexico since 1996 [26] and there are a few studies in Yucatan reporting seroprevalence in urban and rural areas [27, 28], but little is known about the epidemiology of the disease in coastal zones.

The aim of the present work was to determine the seroprevalence of* Ehrlichia* spp. through the indirect immunofluorescence assay test (IFAT), as well as identify associated factors to the presence of antibodies to* Ehrlichia* spp. in dogs from Xcalak, Quintana Roo, Mexico.

## 2. Materials and Methods

### 2.1. Study Area

**Figure 1:**
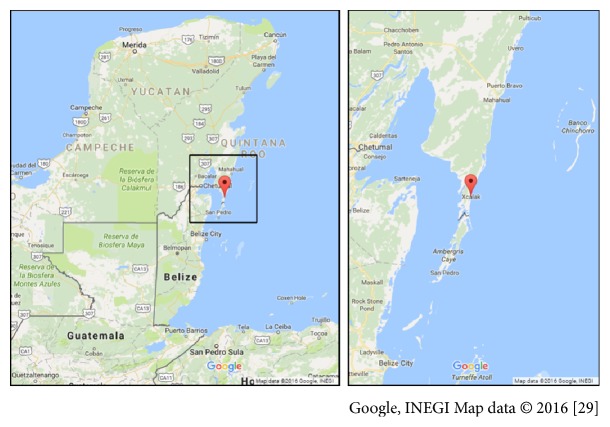
Map showing the geographical position of the studied area.

This study was conducted at the National Park of Xcalak Reefs located in the Southern Coast of Quintana Roo, Mexico, located at latitude 18°30′00′′N and longitude 87°44′49′′W (Figure 1) [29]. The climate is warm and humid, with an annual average temperature of 26.5°C, a minimum of 18°C, and a maximum of 34°C. The annual average rainfall is 1,300 mm [30].

### 2.2. Study Population and Sampling

All population of 118 dogs was sampled in the National Park of Xcalak Reefs, Quintana, Roo, Mexico. Animal handling was performed accordingly to bioethical guidelines to assure their physical integrity. All dogs were physically examined before samples were taken. The owners were interviewed according to a questionnaire in order to obtain information about the dogs. An inspection was done during the physical examination to identify the presence of hemorrhagic signs such as petechiae, ecchymoses, and suffusions, as well as the presence of ticks. Adult ticks were collected from dogs and deposited in plastic containers with 70% ethanol and the tick identification [31] was conducted in the Parasitology Laboratory at the Veterinary Medicine School, UADY, Yucatan, Mexico. Blood samples from each dog were obtained by puncture in the cephalic vein and collected in Vacutainer® tubes with and without EDTA anticoagulant. Tubes without anticoagulant were centrifuged for 5 min at 800 ×g to separate the serum, which was then transferred to 1.5 mL Eppendorf tubes and stored at −20°C until their process in the Laboratory of Immunology at the School of Veterinary Medicine, UADY. A complete cell count, including platelets count and WC counts, was made on the blood samples with EDTA in a semiautomatic impedance analyzer (Sysmex® model KX-21N) at the Small Species Clinic, Veterinary Medicine School, UADY, Yucatan, Mexico.

Less than 200,000 platelets/mL of blood were considered to be thrombocytopenia (26); less than 5.5 million red blood cells/mL, or less than 37% hematocrit, or less than 12 g/dL hemoglobin was considered to be anemia and less than 6,000 white blood cells/mL were considered leucopenia [32].

### 2.3. Detection of Antibodies to* Ehrlichia* spp. 

An indirect immunofluorescent assay test was used to determine IgG antibody titers from serum samples [33]. This assay is considered the reference serological test with a sensitivity of 82 to 100% and a specificity of 67 and 100% [34]. Glass slides containing DH82 cells infected with the Arkansas strain of* E. chaffeensis* (kindly provided by David H. Walker, M.D., from the Department of Pathology at The University of Texas Medical Branch) were used as antigen. Serum samples were diluted in PBS 1 : 100 (pH 7.2) and added to the antigen in the slides. Positive and negative controls were included. Positive control was serum from a dog previously diagnosed with clinical ehrlichiosis and a positive IFAT (1 : 400) confirmed by a positive PCR analysis. Negative control was serum from a dog with no clinical evidence of ehrlichiosis and both negative IFAT and PCR analysis. The glass slides were incubated for 30 minutes at 37°C and then washed three times in PBS (5 minutes each). Rabbit anti-dog IgG labeled with fluorescein isothiocyanate (Sigma-Aldrich Germany) was added in a 1 : 50 PBS dilution and slides were incubated for another 30 minutes at 37°C. After the incubation period, the slides were washed three times in PBS (5 minutes each). Evans blue (0.1% (w/v) in water solution, Sigma-Aldrich, Germany) was added to the last wash buffer as a counterstain. The glass slides were air dried and mounted with buffered glycerol (pH 8.7); then, they were transferred to a microscope equipped for fluorescence (Leica® Germany) and observed at 400x.

### 2.4. Data Analysis

Seroprevalence was calculated using the formula described by Thrusfield [35] for disease occurrence. The independent variables used to determine association with seroprevalence of* Ehrlichia* spp. were age (<1, 1–3, >3 y.o.), ticks (presence, absence), anemia (yes, no), leucopenia (yes, no), thrombocytopenia (yes, no), and hemorrhage (yes, no). Contingency tables (2 × K) were obtained with these variables. Variables with a *p* value ≤ 0.20 were analyzed in SPSS® v. 15.0 (SPSS Inc. 2006) using a binomial logistic regression model with fixed effect to obtain exact estimations of regression, 95% confidence intervals (CI 95%), odds ratio (OR), and *p* value.

## 3. Results

Antibodies against* Ehrlichia* spp. were detected in 64% (75/118) of the dogs. Sixty-one percent of dogs (72/118) were infested with ticks and all ticks were identified as* R. sanguineus s.l*.

The average and standard deviation number of thrombocytes, red blood cells, hemoglobin, hematocrit, and white blood cells in the population were 123,860 +/− 100,041 (range: 12,000–721,000) platelets/mL of blood, 5.36 +/− 1.01 (range: 2.39–7.59) million cells/mL of blood, 41.07 +/− 8.71 (range: 18.9–58.6) percent, and 13,160 +/− 4,180 (range: 3,300–23,800) cells/mL of blood, respectively.

Anemia was found in 36% (43/118) of the dogs, leucopenia in 2.5% (3/118), and thrombocytopenia in 70% (83/118). However, only 14% (17/118) of the dogs presented hemorrhages. Thirty-one percent (23/75) of dogs with anemia, 4% (3/75) of dogs with leucopenia, 80% (60/75) of dogs with thrombocytopenia, 17% (13/75) of dogs with hemorrhages, and 59% (44/75) of dogs with ticks were positive for* Ehrlichia* spp. antibodies. The univariate analysis that was used to identify factors associated with seroprevalence of* Ehrlichia* spp. showed that variables with *p* ≤ 0.20 were age, presence of ticks, thrombocytopenia, and anemia. But the logistic regression indicated a positive association of animals with age 1–3 y.o. (OR = 7.77, CI_95%_ = 2.23–27.04, and *p* = 0.001) and >3 y.o. (OR = 15.39, CI_95%_ = 4.13–57.40, and *p* ≤ 0.001); presence of ticks (OR = 3.13, CI_95%_ = 1.17–8.44, and *p* = 0.023); and thrombocytopenia (OR = 3.36, CI_95%_ = 1.28–8.83, and *p* = 0.013) with seroprevalence of* Ehrlichia* spp. (Table 1).

## 4. Discussion

The epidemiology of canine ehrlichiosis is closely related not only to the vector distribution, which is more frequently associated with tropical and subtropical zones, but also to animal behavior, age, and its environment [27, 36, 37]. Consequently, there is a wide diversity of worldwide seroprevalence. In European countries, such as Bulgaria, Spain, Sweden, and Switzerland, seroprevalence of 30% [38], 19% [36], 17.7% [39], and 2.2% [40], respectively, was reported. In Asia, 14.6% has been reported in Iran [11]; in Africa, 67.8% [41] and 54.2% [42] were recorded in Ivory Coast and Tunisia, respectively.

In America, 44.7% [37] and 63.3% [43] seroprevalence was found in Brazil. In Mexico, Núñez [44] described 33.1% seroprevalence; and in Yucatan, 44.1% [27] and 8.7% [28] were reported. In the present study, a 64% seroprevalence to* Ehrlichia* spp. was found. The wide range of the results reported worldwide might be due to the different serological tests used with different sensitivity and specificity [45]. Also the wide range of seroprevalence found in different countries and regions suggests that environmental conditions associated with the geographical zones play an important role by influencing the tick vector distribution and reproductive cycle [25, 37]. In addition, dogs that are 1–3 and >3 y.o. had 7.77–15.39 chances of being serologically positive. Similar results have been reported in Yucatan, Mexico (2–4 y.o.: OR = 6.77, *p* = 0.005; >4 y.o.: OR =4.24, *p* = 0.043), and Brazil (2–5 y.o.: OR = 2.20, *p* ≤ 0.05; >5 y.o.: OR = 2.96, *p* ≤ 0.01), by Rodriguez-Vivas et al. [27] and Costa et al. [37], respectively. This trend could be explained by the fact that older animals have a bigger chance of being exposed to vectors than younger animals. As a consequence, the chances of being exposed to the etiological agent of ehrlichiosis increase. Baneth et al. [46] have shown a higher susceptibility to ehrlichiosis in adult dogs. On the other hand, antibody titers remain during prolonged periods, even when the patient has a full clinical recovery [3, 5, 6]. The presence of ticks was another factor associated with the seroprevalence (OR = 3.13, CI 95% = 1.17–8.44, and *p* = 0.023), similar to the report by Costa et al. [37] (OR = 2.1, *p* ≤ 0.01) in Brazil. All the ticks in this study were identified as* R. sanguineus* and a 61% infestation was observed on the dogs. The presence of the vector is essential for the transmission of the disease in the epidemiology of ehrlichiosis as noted by Harrus et al. [5], Skotarczak [20], and Stich et al. [22]. Several studies have shown that the prevalence of ticks and the seroprevalence to* Ehrlichia* spp. in Mexico vary accordingly to geographical zones. Núñez [44] reported seroprevalence of 48.3%, 24.1%, and 70.2%, to* E. canis* in the States of Sinaloa, Morelos, and Baja California Mexico, respectively. Also, 46% [47], 20% [48], and 59.6% [49] prevalence of* R. sanguineus* has been found in those same states. Herein, dogs presenting thrombocytopenia had 3.36 more chances of having a positive antibody titer. Other authors, such as Rodriguez-Vivas et al. [27], found that dogs had 18.91 more chances of having a positive antibody titer when presenting thrombocytopenia. This evidence confirms that thrombocytopenia is the most common and consistent clinical sign of canine ehrlichiosis [5, 7, 50, 51]. Niwetpathomwat et al. [52] found, in a retrospective study involving 687 cases with a diagnosis of canine ehrlichiosis, that thrombocytopenia was present in 93% of the dogs. In addition, the three species of* Ehrlichia* spp. (*E. canis*,* E. chaffeensis*, and* E. ewingii*) that can be infecting the studied population produce thrombocytopenia [2, 20, 53, 54].

*R. sanguineus s.l.* on dogs has a wide distribution in the Yucatan Peninsula [31] and this tropical lineage of* R. sanguineus* has showed to be a competent vector for* E. canis* [55]. Pat-Nah et al. [56] found in Yucatan, Mexico, that* R. sanguineus s.l.* ticks are the vector of* E. canis* infections in dogs. The high infestations of dogs and the wide distribution of* R. sanguineus s.l.* on dogs in the Yucatan Peninsula highlight the risk for* Ehrlichia* transmission [31].

The results in this study show that antibodies to* Ehrlichia* spp. are present in the dogs of Xcalak, Quintana Roo, Mexico; however, further molecular studies are required to identify which species of the genogroup (*E. canis*,* E. chaffeensis*, and* E. ewingii*) are present in this dog population. This is the first report of dog's exposure to* Ehrlichia* spp. in the coastal zone of the Yucatan Peninsula.

## 5. Conclusion

It is concluded that the seroprevalence of* Ehrlichia* spp. in the dog population of Quintana Roo coast is high, and therefore the disease can be considered endemic to the region. The associated factors are increased age, tick infestation, and thrombocytopenia.

## Figures and Tables

**Table 1:** Logistic regression analysis to detect associated factors to anti-*Ehrlichia *spp. antibody response in 118 dogs from Xcalak, Quintana Roo, Mexico.

Variable	Total	Positive	Prevalence (%)	OR	CI_95%_	*p *value
Age						
<1 year	29	8	28			
1–3 years	42	30	71	7.77	2.23–27.04	**0.001**
>3 years	47	37	78	15.39	4.13–57.40	**<0.001**
Ticks						
No	57	31	54			
Yes	61	44	72	3.13	1.17–8.41	**0.023**
Thrombocytopenia						
No	35	15	43			
Yes	83	60	72	3.36	1.28–8.83	**0.013**
Anemia						
No	75	52	69			
Yes	43	23	53	0.93	0.32–2.70	0.891

OR: odds ratio; CI_95%_: confidence interval (95%); *p *value: probability.
